# Unraveling the Role of Toll-like Receptors in the Immunopathogenesis of Selected Primary and Secondary Immunodeficiencies

**DOI:** 10.3390/cells12162055

**Published:** 2023-08-12

**Authors:** Paulina Mertowska, Konrad Smolak, Sebastian Mertowski, Ewelina Grywalska

**Affiliations:** Department of Experimental Immunology, Medical University of Lublin, 20-093 Lublin, Poland

**Keywords:** CLL, CVID, TLR, SID, PID, immunodeficiency

## Abstract

The human immune system is a complex network of cells, tissues, and molecules that work together to defend the body against pathogens and maintain overall health. However, in some individuals, the immune system fails to function correctly, leading to immunodeficiencies. Immunodeficiencies can be classified into primary (PID) and secondary (SID) types, each with distinct underlying causes and manifestations. Toll-like receptors (TLRs), as key components of the immune system, have been implicated in the pathogenesis of both PID and SID. In this study, we aim to unravel the intricate involvement of TLR2, TLR4, TLR3, TLR7, TLR8, and TLR9 in the immunopathogenesis of common variable immunodeficiency—CVID (as PID)—and chronic lymphocytic leukemia—CLL (as SID). The obtained results indicate a significant increase in the percentage of all tested subpopulations of T lymphocytes and B lymphocytes showing positive expression of all analyzed TLRs in patients with CVID and CLL compared to healthy volunteers, constituting the control group, which is also confirmed by analysis of the concentration of soluble forms of these receptors in the plasma of patients. Furthermore, patients diagnosed with CVID are characterized by the percentage of all lymphocytes showing positive expression of the tested TLR2, TLR4, TLR3, and TLR9 and their plasma concentrations in relation to patients with CLL. By investigating the functions and interactions of TLRs within the immune system, we seek to shed light on their critical role in the development and progression of these immunodeficiencies. Through a comprehensive analysis of the literature and presented experimental data, we hope to deepen our understanding of the complex mechanisms by which TLRs contribute to the pathogenesis of PID and SID. Ultimately, our findings may provide valuable insights into developing targeted therapeutic strategies to mitigate the impact of these disorders on those affected by immunodeficiency.

## 1. Introduction

The human immune system is crucial in defending the body against pathogens and maintaining overall health [1]. However, in some individuals, the immune system fails to function optimally, leading to primary (PID) and secondary immunodeficiency (SID) [2]. These conditions are characterized by an increased susceptibility to infections and a heightened risk of developing severe complications. Understanding the underlying mechanisms responsible for the pathogenesis of immunodeficiencies is crucial for the developing therapeutic interventions [3,4,5]. PIDs are typically caused by genetic defects that affect immune cells’ or molecules’ development or function. These defects can lead to a compromised immune response, making individuals more susceptible to infections. On the other hand, secondary immunodeficiencies arise from external factors that impair the immune system’s ability to fight off infections. These factors can include underlying medical conditions, such as HIV/AIDS, cancer, autoimmune diseases, and certain medications or treatments.

Among the various components of the immune system, Toll-like receptors (TLRs) have emerged as key players in recognizing and initiating immune responses against invading microorganisms [6,7,8]. TLRs are a family of pattern recognition receptors (PRRs) that recognize specific molecular patterns associated with pathogens, known as pathogen-associated molecular patterns (PAMPs). Upon activation, TLRs trigger a cascade of signaling events that activate the immune response, including producing pro-inflammatory cytokines and recruiting immune cells to the site of infection [9,10]. Dysregulation of TLR signaling pathways has been implicated in certain PIDs, contributing to impaired immune cell activation and compromised pathogen recognition [11,12,13].

TLRs are also involved in the immunopathogenesis of SIDs by mediating the inflammatory response and regulating immune cell function. Altered TLR signaling in these conditions can disrupt the delicate balance of immune responses, leading to increased susceptibility to infections or impaired immune surveillance [14,15,16]. In recent years, growing evidence has implicated TLRs in the pathogenesis of both PIDs and SIDs. Aberrant TLR signaling has been associated with dysregulated immune responses, impaired immune cell function, and defective immune system development. Understanding the precise role of TLRs in these immunodeficiencies could provide valuable insights into disease mechanisms and potentially open new avenues for therapeutic interventions [1].

This study aims to unravel the role of Toll-like receptors in the immunopathogenesis of carefully selected primary and secondary immunodeficiencies, exemplified by common variable immunodeficiency (CVID) and chronic lymphocytic leukemia (CLL). The analyses carried out are aimed at determining changes in the percentage of T and B lymphocyte subpopulations in the peripheral blood with positive expression of TLR2, TLR3, TLR4, TLR7, TLR8, and TLR9 receptors, as well as the concentration of their soluble forms in the serum of patients with CVID and CLL in relation to healthy volunteers, constituting the control group.

CVID is a PID disorder characterized by a deficiency in the production of antibodies. It is considered one of the most common primary immunodeficiencies, hence the name [17]. The primary feature of CVID is a decreased production of antibodies, specifically immunoglobulins, which are essential for fighting off infections. This deficiency increases susceptibility to recurrent bacterial, viral, and fungal infections, particularly in the respiratory and gastrointestinal tracts [18]. CVID typically manifests in late childhood, adolescence, or adulthood. The age of onset can vary, but most individuals are diagnosed between the ages of 20 and 40 [19]. The symptoms and severity of CVID can vary widely among individuals. Some people may experience frequent infections, while others may have more subtle symptoms or are asymptomatic. This variability makes diagnosis challenging and often leads to delayed recognition. While the primary defect in CVID lies in antibody production, some individuals with CVID may also have abnormalities in T cells, another type of immune cell. T cells are crucial in coordinating the immune response and directly killing infected cells. Abnormalities in T cell function can further weaken the immune system and contribute to the susceptibility to infections seen in CVID [20,21]. The immune dysregulation in CVID can lead to chronic inflammation and an increased risk of autoimmune disorders. The imbalance in the immune response can cause the immune system to mistakenly attack the body’s tissues and organs, resulting in autoimmune conditions such as rheumatoid arthritis, lupus, or inflammatory bowel disease [22].

CLL is a type of cancer affecting a specific white blood cell type called lymphocytes. It is characterized by the abnormal and uncontrolled growth of these lymphocytes, particularly B cells, in the bone marrow, blood, and lymph nodes. These lymphocytes are often mature but do not function properly, leading to a weakened immune system. CLL is generally a slow-progressing form of leukemia [23,24,25]. The abnormal lymphocytes divide and accumulate over time, gradually replacing healthy cells in the bone marrow. Many individuals with CLL may not have symptoms in the early stages, and the disease is often detected incidentally during routine blood tests [26,27]. CLL weakens the immune system, making individuals more prone to infections. The abnormal lymphocytes fail to effectively combat infections, leading to recurrent respiratory tract infections. These infections can be more severe and last longer than usual [28,29]. A subset of CLL patients may develop hypogammaglobulinemia or low levels of immunoglobulins (antibodies) in the blood. This can further compromise the immune system’s ability to fight off infections. CLL can also affect T cell function, although to a lesser extent than B cell dysfunction. T cells play a critical role in cell-mediated immunity, helping to recognize and destroy infected or cancerous cells. The interaction between CLL cells and T cells and the immunosuppressive microenvironment created by CLL can lead to T cell dysfunction, reducing the immune response [30,31,32].

## 2. Materials and Methods

### 2.1. Research Material and Characteristics of Patients Included in The study

The research material consisted of 10 mL of patients’ peripheral blood collected from the basilic vein for EDTA-containing samples and 5 mL of blood collected in a clot tube to obtain serum for further analysis. Peripheral whole blood was used for the immunophenotypic analysis and the assessment of the percentage of occurrence of individual subpopulations of T and B lymphocytes with positive expression of all tested TLRs.

The group of patients consisted of 40 people with newly diagnosed CVID and 40 people with newly diagnosed CLL. All immunodeficient patients were subject to the inclusion and exclusion criteria. Patient selection was performed by a physician experienced in clinical immunology, based on specific criteria: age ≥ 18 years; life expectancy ≥ 12 months; no immunosuppressive treatment within 3 months before study entry; written consent to participate in the study. Criteria for exclusion of patients from the study: active viral, bacterial, or fungal infection; severe allergy; condition after allotransplantation of hematopoietic cells or internal organs; active malignancy or another autoimmune disease under treatment; pregnancy or lactation; taking drugs that are in clinical trials; presence of tumor metastases within the central nervous system or mental illness. The control group consisted of 40 healthy volunteers who were also subject to the same inclusion and exclusion criteria as the patients in the study group. All patients were age- and gender-matched: CVID (median age: 46.1; range: 27–69; 15 females and 25 males); CLL (median age: 45.2; range: 31–63; 17 females and 23 males); healthy volunteers (median age: 46.8; range: 32–70; 18 females and 22 males). The study protocol was positively evaluated by the Bioethics Committee at the Medical University of Lublin—KE-0254/276/2021.

### 2.2. Peripheral Blood Immunophenotype Analysis

**Figure 1:**
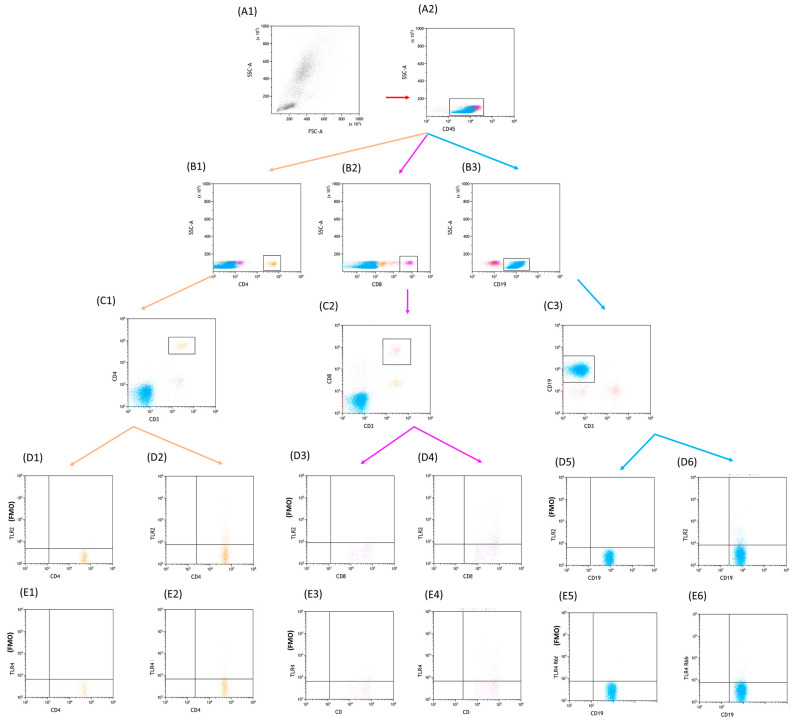
Sample analysis and gating strategy employed to identify specific lymphocyte populations in CLL patients. The focus of the analysis is on CD4+CD8+TLR2+ and CD4+CD8+TLR4+ T lymphocytes, as well as CD19+TLR2+ and CD19+TLR4+ B lymphocytes. Panels (**A1**) and (**A2**) presents the gating strategy used to identify CD45-positive cells, which serves as an initial step in the analysis. Moving forward, panels (**B1**) and (**C1**) showcase the identification of CD3+CD4+ lymphocytes, while panels (**B2**) and (**C2**) demonstrate the gating strategy for CD3+CD8+ lymphocytes. Panels (**B3**) and (**C3**) illustrate the gating strategy for CD3-CD19+ lymphocytes. To further explore the analysis, panels (**D1**) and (**D2**) depict the identification of CD4+TLR2+ lymphocytes, while panels (**D3**) and (**D4**) highlight the gating strategy for CD8+TLR2+ lymphocytes. Panels (**D5**) and (**D6**) demonstrate the gating strategy employed for CD19+TLR2+ lymphocytes. Moreover, panels (**E1**) and (**E2**) provide insight into the identification of CD4+TLR4+ lymphocytes. Similarly, panels (**E3**) and (**E4**) exhibit the gating strategy for CD8+TLR4+ lymphocytes, while panels (**E5**) and (**E6**) showcase the gating strategy used to identify CD19+TLR4+ lymphocytes. (**D1**, **E1**, **D3**, **E3**, **D5**, **E5**—FMO control). Blue: CD3+CD4+, violet: CD3+CD8+, orange: CD3-CD19+, red: CD45+.

**Figure 2:**
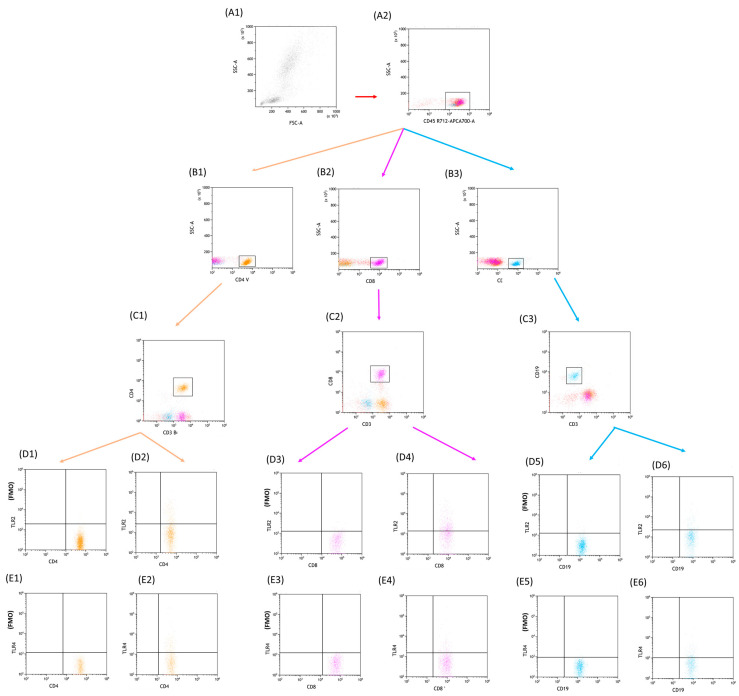
Sample analysis and gating strategy employed to identify specific lymphocyte populations in CVID patients. The focus of the analysis is on CD4+CD8+TLR2+ and CD4+CD8+TLR4+ T lymphocytes, as well as CD19+TLR2+ and CD19+TLR4+ B lymphocytes. Panels (**A1**) and (**A2**) present the gating strategy used to identify CD45-positive cells, which serves as an initial step in the analysis. Moving forward, panels (**B1**) and (**C1**) showcase the identification of CD3+CD4+ lymphocytes, while panels (**B2**) and (**C2**) demonstrate the gating strategy for CD3+CD8+ lymphocytes. Panels (**B3**) and (**C3**) illustrate the gating strategy for CD3−CD19+ lymphocytes. To further explore the analysis, panels (**D1**) and (**D2**) depict the identification of CD4+TLR2+ lymphocytes, while panels (**D3**) and (**D4**) highlight the gating strategy for CD8+TLR2+ lymphocytes. Panels (**D5**) and (**D6**) demonstrate the gating strategy employed for CD19+TLR2+ lymphocytes. Moreover, panels (**E1**) and (**E2**) provide insight into the identification of CD4+TLR4+ lymphocytes. Similarly, panels (**E3**) and (**E4**) exhibit the gating strategy for CD8+TLR4+ lymphocytes, while panels (**E5**) and (**E6**) showcase the gating strategy used to identify CD19+TLR4+ lymphocytes. (**D1**, **E1**, **D3**, **E3**, **D5**, **E5**—FMO control). Blue: CD3+CD4+, violet: CD3+CD8+, orange: CD3−CD19+, red: CD45+.

Flow cytometry was employed to examine the immunophenotype of lymphocytes found in peripheral blood. The process involved obtaining a whole blood sample, which was then treated with a set of monoclonal human antibodies comprising anti-CD4 BV421, anti-CD3 PerCp, anti-CD8 BV605, anti-CD19 FITC, anti-CD45 Alexa Fluor 700, anti-CD56 BV650, anti-CD16 BV650, anti-TLR-2 APC, anti-TLR-4 PE, anti-TLR-7 PE, anti-TLR-8 APC, anti-TLR-3 PE, and anti-TLR-9 APC (Biolegend, San Diego, CA, United States). Following the antibody staining, a lysing buffer (BD, Franklin Lakes, NJ, United States) was applied to eliminate red blood cells, and the resulting cells were thoroughly washed and evaluated using a CytoFLEX LX instrument (Beckman Coulter, Indianapolis, IN, United States). Subsequently, data analysis was carried out utilizing the Kaluza Analysis program (sample analysis in Figure 1—CLL and Figure 2—CVID) The CytoFLEX LX flow cytometer was internally quality controlled using CytoFLEX Ready to Use Daily QC Fluorosphere reagents (Beckman Coulter, Indianapolis, IN, United States).

### 2.3. Evaluation of the Concentration of Soluble Forms of the Tested TLRs in the Serum of Patients Included in the Study

The research material for the assessment of the concentration of soluble forms of TLR by means of enzyme immunoassays (ELISA) was the serum collected from all patients included in the study. The assays were carried out using commercially available kits in accordance with the manufacturer’s instructions: Human TLR2 ELISA Kit (sensitivity: 17 pg/mL; range: 109.4–7000 pg/mL); Human TLR3 ELISA Kit (sensitivity: 10 pg/mL; range: 156–10,000 pg/mL); Human TLR4 ELISA Kit (sensitivity: 0.4 ng/well; range: 0.41–100 ng/mL) (Abcam, Cambridge, UK); HumanToll Like Receptor 7 (TLR7) ELISA Kit (sensitivity: 32.6 pg/mL; range: 78.1–5000 pg/mL); Human Toll-Like Receptor 8 (TLR-8) ELISA Kit (sensitivity: 0.06 ng/mL; range: 20–0.312 ng/mL); Human Toll-Like Receptor 9 (TLR-9) ELISA Kit (sensitivity: 0.06 ng/mL; range: 20–0.312 ng/mL) (MyBiosource, San Diego, CA, USA). The measurement was performed using a VictorTM3 reader (PerkinElmer, Waltham, MA, USA).

### 2.4. Statistical Analysis of the Results Obtained

Statistical analysis of the obtained results was carried out using Tibco Statistica 13.3 software (Palo Alto, CA, USA). The assessment of the normality of the data distribution was determined using the Shapiro–Wilk test. Differences between the groups were analyzed using the Kruskal–Wallis test followed by Dunn’s post hoc test; *p*-values for Dunn’s test were adjusted for multiple comparisons with the Bonferroni method. Spearman correlation coefficients were used to study relationships between pairs of variables. In addition, the diagnostic performance of the laboratory test was determined using ROC curves for patient-related parameters. Visualizations of the obtained data were performed using GraphPad Prism (GraphPad Prism Software v. 9.4.1 (687), Boston, MA, USA).

## 3. Results

### 3.1. The Importance of Peripheral Blood Counts and Immunoglobulin Levels in Immunodeficient Patients Compared to Healthy Volunteers

A change in peripheral blood counts and immunoglobulin levels in the serum of immunodeficient patients is one of the characteristic changes. Immunoglobulins are responsible for recognizing and neutralizing pathogens, thereby protecting the body against infections. In the case of patients with CVID and CLL included in our study, a significant decrease in the levels of antibodies in the IgG and IgA class was also observed compared to healthy volunteers (Table 1). In individuals with CVID and CLL, there is often a decrease in one or more classes of immunoglobulins, such as IgG, IgA, and/or IgM. These reduced levels can lead to an impaired immune response and an increased susceptibility to infections. In addition, a detailed analysis of peripheral blood counts showed a number of statistically significant changes in the number of white blood cells, lymphocytes, neutrophils, and red blood cells, as well as hemoglobin values (Table 1). The noted differences were significant not only in relation to healthy volunteers but also between patients with CVID and CLL (Table 1).

### 3.2. Analysis of the Immunophenotype in the Course of Immunodeficiency in Patients with CVID and CLL in Relation to Healthy Volunteers

In the context of immunodeficiencies, understanding the state of the immune system during disease requires an in-depth analysis of the immunophenotype, which refers to the specific characteristics and composition of the immune cells present in a person’s immune system. Immunophenotyping involves the identification and quantification of different subsets of immune cells, such as T cells, B cells, and natural killer (NK) cells. Analysis of the immunophenotype of patients with CVID and CLL showed a number of significant changes in the percentage of all immune cell populations tested (Table 2). Particularly noteworthy is the observed decrease in the number of CD3+ and CD4+CD3+ T lymphocytes, both in patients with CVID and CLL compared to healthy volunteers (Table 2). In the case of patients with CLL, we also noted a significant increase in the percentage of CD19+ B cells compared to the other groups of patients and, in patients with CVID, an additional increase in the percentage of CD8+CD3+ T cells (Table 2). A detailed analysis of the peripheral blood immunophenotype of patients with immunodeficiencies provides insight into the state of the immune system and its changes during the progression or treatment of individual immunodeficiencies.

### 3.3. Investigation of the Percentage of Selected Subpopulations of Peripheral Blood Lymphocytes Showing Positive Expression of Tested TLRs in Immunodeficient Patients Compared to Healthy Volunteers

**Figure 3:**
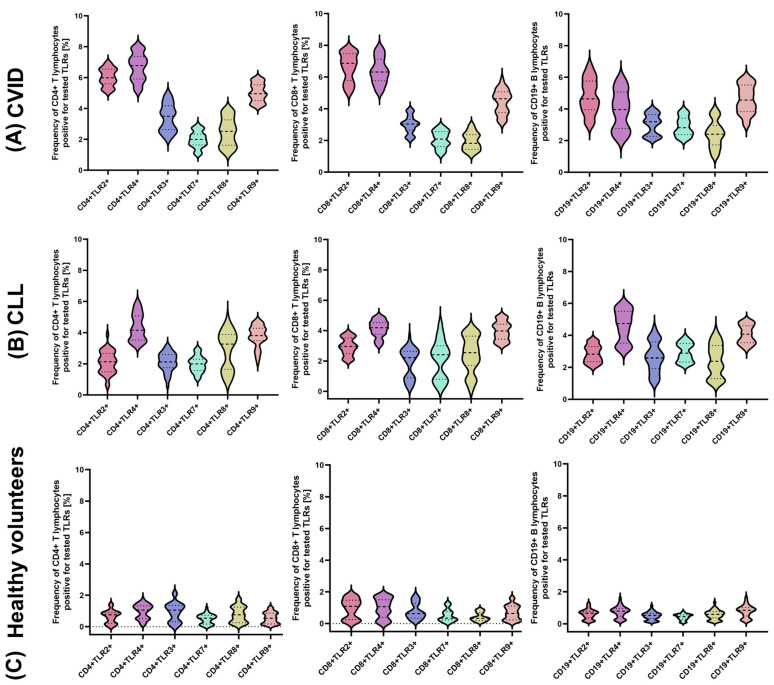
Analysis of the percentage of T and B lymphocyte subpopulations positive for TLR2, TLR4, TLR3, TLR7, TLR8, and TLR9 in selected immunodeficient patients and healthy volunteers. (**A**) Analysis of the percentage of T and B cell subpopulations positive for TLR2, TLR4, TLR3, TLR7, TLR8, TLR9 in patients with CVID; (**B**) analysis of the percentage of T and B lymphocyte subpopulations positive for TLR2, TLR4, TLR3, TLR7, TLR8, TLR9 in CLL patients; (**C**) analysis of the percentage of T and B cell subpopulations positive for TLR2, TLR4, TLR3, TLR7, TLR8, TLR9 in healthy volunteers.

The next stage of our analysis was the assessment of the percentage of occurrence of individual subpopulations of peripheral blood lymphocytes with positive expression of TLR2, TLR4, TLR3, TLR7, TLR8, and TLR9 receptors. The obtained results are presented in Figure 3 and Table 3.

The bold dashed line indicates the median, the two thinner dashed lines indicate the results in the range of 25 and 75%. The ends of the figures on the graph mean the minimum and maximum in the data range.

**Figure 4:**
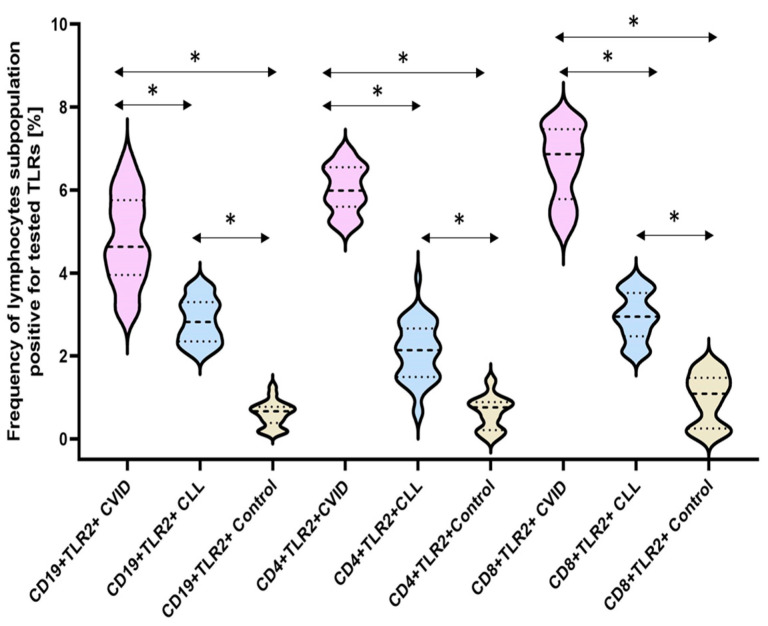
Schematic presentation of the differences between the percentage of occurrence of individual T and B lymphocyte subpopulations showing positive expression of TLR2, taking into account the disease entities. * Statistically significant results. In order to better emphasize the existing differences, different groups of patients are marked with a different color. Patients diagnosed with CVID are marked with a light purple color, patients with CLL with a light blue color, and patients from the control group, i.e., healthy volunteers, with a beige color. The bold dashed line indicates the median, the two thinner dashed lines indicate the results in the range of 25 and 75%. The ends of the figures on the graph mean the minimum and maximum in the data range.

In the case of the TLR2 receptor, the analysis showed a significant increase in the percentage of all tested subpopulations of T and B lymphocytes positively expressing this receptor in patients with immunodeficiency compared to healthy volunteers. This increase was 9.41-fold higher for CD4+TLR2+ patients, 6.91-fold higher for CD8+TLR2+, and 8-fold higher for CD19+TLR2+ compared to the control group (Figure 4) (Table 3). In contrast, in patients with CLL, the increase was 3.28-fold for CD4+TLR2+, 3.07-fold for CD8+TLR2+, and 4.75-fold for CD19+TLR2+ relative to the control group (Figure 4) (Table 3). In addition, we showed statistically significant differences between CVID and CLL in the case of the percentage of occurrence of all tested immune cell subpopulations showing positive expression of TLR2 (Table 3).

**Figure 5:**
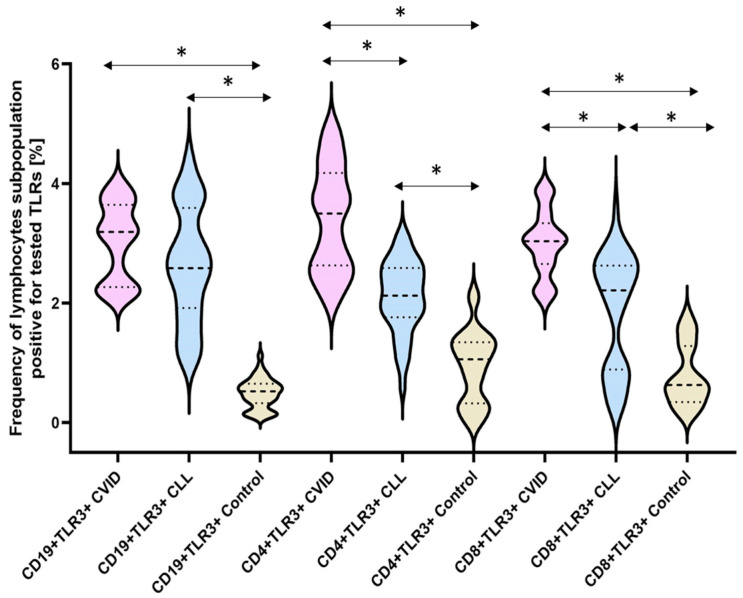
Schematic presentation of the differences between the percentage of occurrence of individual T and B lymphocyte subpopulations showing positive expression of TLR3, taking into account the disease entities. * Statistically significant results. In order to better emphasize the existing differences, different groups of patients are marked with a different color. Patients diagnosed with CVID are marked with a light purple color, patients with CLL with a light blue color, and patients from the control group, i.e., healthy volunteers, with a beige color. The bold dashed line indicates the median, the two thinner dashed lines indicate the results in the range of 25 and 75%. The ends of the figures on the graph mean the minimum and maximum in the data range.

The analysis of the percentage of TLR3-positive lymphocytes showed a statistically significant increase in all immunodeficient patients compared to healthy volunteers. For patients with CVID, it was: 3.61-fold for CD4+TLR3+, 3.78-fold for CD8+TLR3+, and 6.18-fold for CD19+TLR3+. In patients with CLL, this increase was slightly lower than in patients with CVID and was, respectively: 2.24-fold for CD4+TLR3+, 2.39-fold for CD8+TLR3+, and 5.38-fold for CD19+TLR3+ (Figure 5) (Table 3).

**Figure 6:**
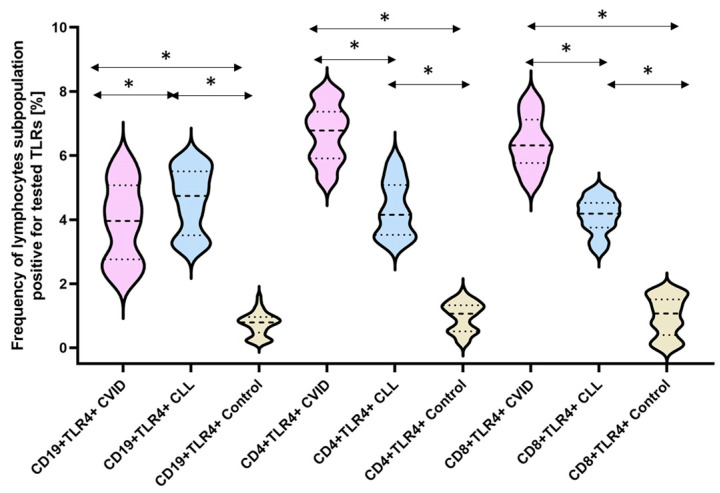
Schematic presentation of the differences between the percentage of occurrence of individual T and B lymphocyte subpopulations showing positive expression of TLR4, taking into account the disease entities. * Statistically significant results. In order to better emphasize the existing differences, different groups of patients are marked with a different color. Patients diagnosed with CVID are marked with a light purple color, patients with CLL with a light blue color, and patients from the control group, i.e., healthy volunteers, with a beige color. The bold dashed line indicates the median, the two thinner dashed lines indicate the results in the range of 25 and 75%. The ends of the figures on the graph mean the minimum and maximum in the data range.

One of the highest observed values was recorded for TLR4. The mean values and percentages of this receptor’s occurrence on individual subpopulations of immune cells tested in patients with CVID were: 6.84-fold for CD4+TLR4+, 6.53-fold for CD8+TLR4+, and 6.22-fold for CD19+TLR4+, while in patients with CLL they were 4.43-fold, 4.20-fold, and 5.30-fold the values observed in healthy volunteers, respectively (Figure 6) (Table 3).

**Figure 7:**
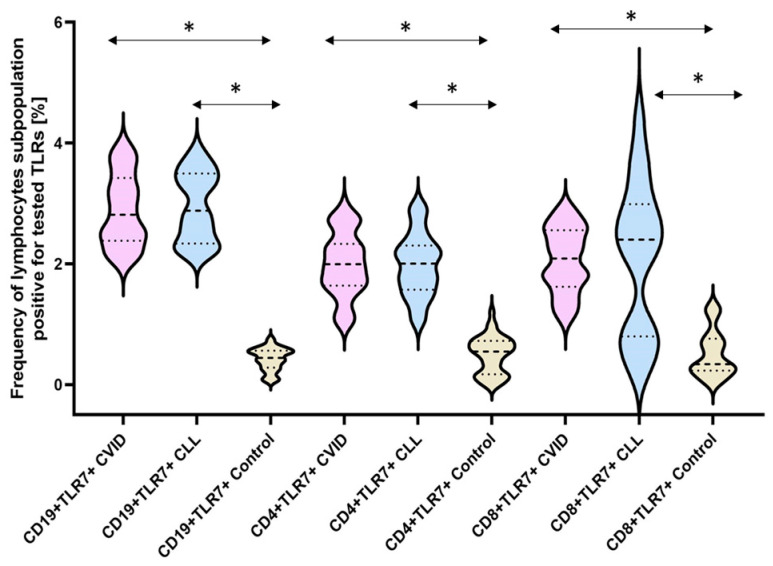
Schematic presentation of the differences between the percentage of occurrence of individual T and B lymphocyte subpopulations showing positive expression of TLR7, taking into account the disease entities. * Statistically significant results. In order to better emphasize the existing differences, different groups of patients are marked with a different color. Patients diagnosed with CVID are marked with a light purple color, patients with CLL with a light blue color, and patients from the control group, i.e., healthy volunteers, with a beige color. The bold dashed line indicates the median, the two thinner dashed lines indicate the results in the range of 25 and 75%. The ends of the figures on the graph mean the minimum and maximum in the data range.

**Figure 8:**
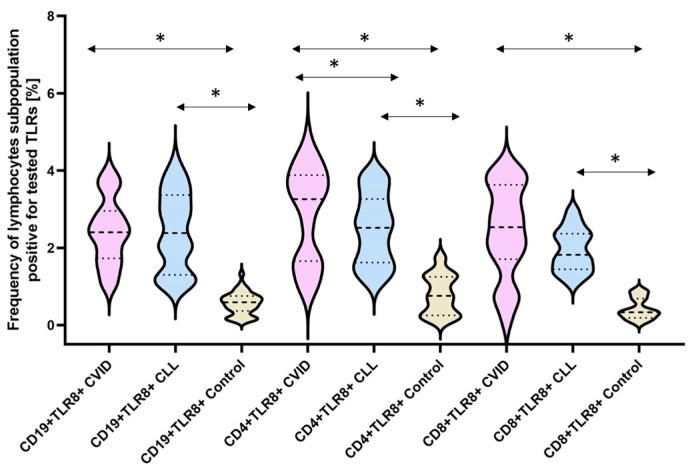
Schematic presentation of the differences between the percentage of occurrence of individual T and B lymphocyte subpopulations showing positive expression of TLR8, taking into account the disease entities. * Statistically significant results. In order to better emphasize the existing differences, different groups of patients are marked with a different color. Patients diagnosed with CVID are marked with a light purple color, patients with CLL with a light blue color, and patients from the control group, i.e., healthy volunteers, with a beige color. The bold dashed line indicates the median, the two thinner dashed lines indicate the results in the range of 25 and 75%. The ends of the figures on the graph mean the minimum and maximum in the data range.

In the case of the next two analyzed TLRs: TLR7 and TLR8, we noted a statistically significant increase in the percentage of all tested lymphocyte subpopulations showing their positive expression in both CVID and CLL patients compared to the control group (Figure 7 and Figure 8) (Table 3). However, we did not find statistically significant differences between the two types of immunodeficiency, with the exception of CD8+TLR8+ (Figure 8) (Table 3).

**Figure 9:**
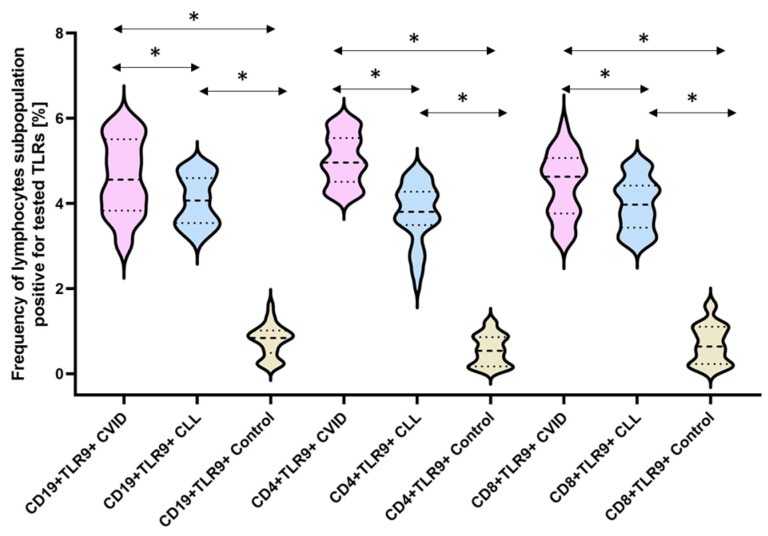
Schematic presentation of the differences between the percentage of occurrence of individual T and B lymphocyte subpopulations showing positive expression of TLR9, taking into account the disease entities. * Statistically significant results. In order to better emphasize the existing differences, different groups of patients are marked with a different color. Patients diagnosed with CVID are marked with a light purple color, patients with CLL with a light blue color, and patients from the control group, i.e., healthy volunteers, with a beige color. The bold dashed line indicates the median, the two thinner dashed lines indicate the results in the range of 25 and 75%. The ends of the figures on the graph mean the minimum and maximum in the data range.

The last analyzed TLR9 showed a statistically significant increase in the percentage of analyzed subpopulations of lymphocytes showing its positive expression both in patients with CVID (9.14 times for CD4+TLR9+; 6.43 times for CD8+TLR9+; and 5.91 times for CD19 +TLR9+) and CLL (6.84-fold for CD4+TLR9+; 5.77-fold for CD8+TLR9+; and 5.20-fold for CD19+TLR9+) in relation to a group of healthy volunteers, as well as among themselves (Figure 9) (Table 3).

### 3.4. Quantitative Analysis of Soluble Forms of TLRs (sTLRs) in the Serum of Immunodeficient Patients in Relation to Healthy Volunteers

In the next stage of the research, an analysis of the serum concentration of soluble TLRs from the tested receptors was performed. As can be seen in Table 4, patients with diagnosed immunodeficiencies were characterized by higher concentrations of all analyzed TLRs compared to healthy volunteers.

**Figure 10:**
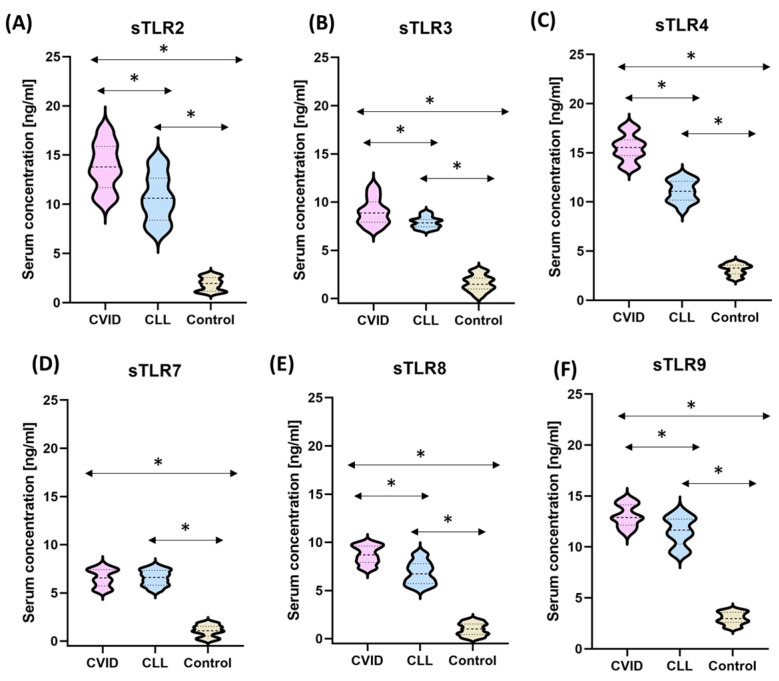
The serum concentration of soluble forms of tested TLRs (sTLRs), including values for individual disease entities and healthy volunteers. (**A**) Serum sTLR2 concentrations of patients with CVID, CLL, and healthy volunteers; (**B**) serum sTLR3 concentrations of patients with CVID, CLL, and healthy volunteers; (**C**) serum sTLR4 concentrations of CVID, CLL patients, and healthy volunteers; (**D**) serum sTLR7 concentrations of CVID, CLL patients, and healthy volunteers; (**E**) serum sTLR8 concentrations of CVID, CLL patients and healthy volunteers; (**F**) serum sTLR9 concentrations of CVID, CLL patients, and healthy volunteers. * Statistically significant results. In order to better emphasize the existing differences, different groups of patients are marked with a different color. Patients diagnosed with CVID are marked with a light purple color, patients with CLL with a light blue color, and patients from the control group, i.e., healthy volunteers, with a beige color. The bold dashed line indicates the median, the two thinner dashed lines indicate the results in the range of 25 and 75%. The ends of the figures on the graph mean the minimum and maximum in the data range.

Moreover, detailed analysis showed that, with the exception of sTLR7, all serum concentrations of the tested TLRs are higher in patients with CVID than in CLL (Figure 10A–F) (Table 4). The highest values relative to healthy volunteers were observed for sTLR2 and sTLR4 concentrations, being above 13 ng/mL and 15 ng/mL for CVID and above 10 ng/mL and 11 ng/mL for CLL, respectively. However, these values were not the largest difference observed compared to healthy volunteers (7.32-fold and 4.96-fold for CVID patients and 5.64-fold and 3.54-fold for CLL patients) (Figure 10A,C). The highest increase relative to healthy subjects was observed for sTLR8, the difference of which was 8.96-fold for CVID and 7.02-fold for CLL, respectively (Figure 10E). For sTLR3, the changes were 5.73-fold higher for CVID and 5.01-fold higher for CLL (Figure 10B), while for sTLR9, the changes were 4.35-fold and 3.85-fold higher (Figure 10F).

### 3.5. The Role of Tested TLRs in the Immunopathogenesis of Immunodeficiencies—Analysis of Correlations

**Figure 11:**
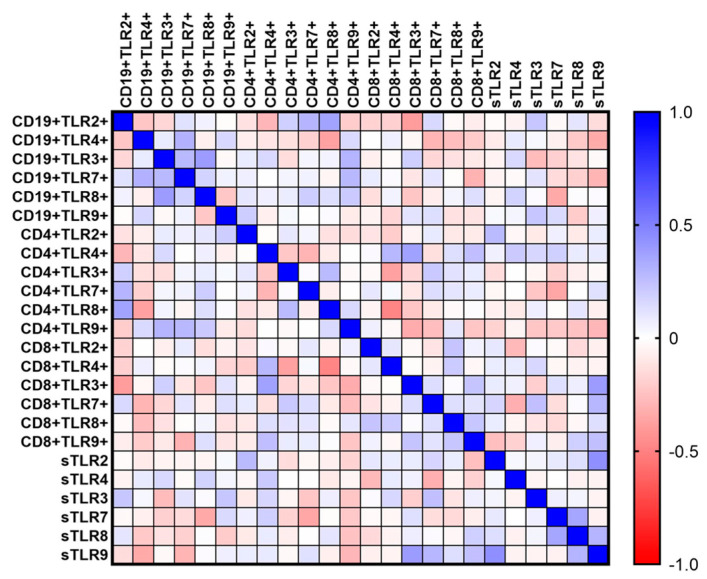
Schematic representation of Spearman rank correlation analysis for selected immunological parameters in patients with CVID. Negative correlations are marked in red and positive correlations in blue.

**Figure 12:**
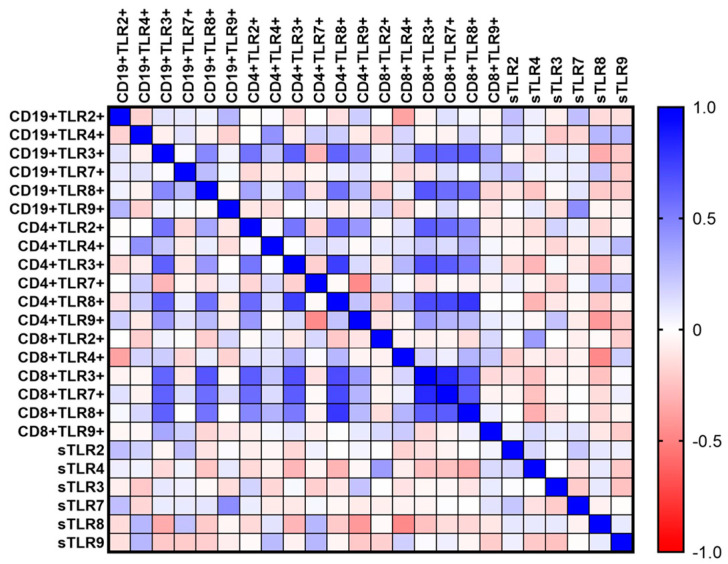
Schematic representation of Spearman rank correlation analysis for selected immunological parameters in patients with CLL. Negative correlations are marked in red and positive correlations in blue.

In the next stage, the results of the study were used to analyze the correlation between the percentage of occurrence of individual subpopulations of T and B lymphocytes with positive expression of TLRs in relation to the concentration of their sTLRs in the serum. This analysis was performed for both CVID patients (Figure 11) (Table 5) and CLL patients (Figure 12) (Table 6).

In the case of both analyzed types of immunodeficiencies, positive correlations prevail. In the case of patients with CVID, these are low positive correlations, while in patients with CLL, not only low but also moderate or high positive correlations have been reported. The obtained results indicate that TLRs form a signaling network of cells of the immune system, which are involved in the dysregulation of the proper functioning of the immune response and thus also in the immunopathogenesis of immunodeficiencies.

### 3.6. The Potential of TLRs as Predictive Biomarkers for the Detection of Immunodeficiencies—ROC Curve Analysis

**Figure 13:**
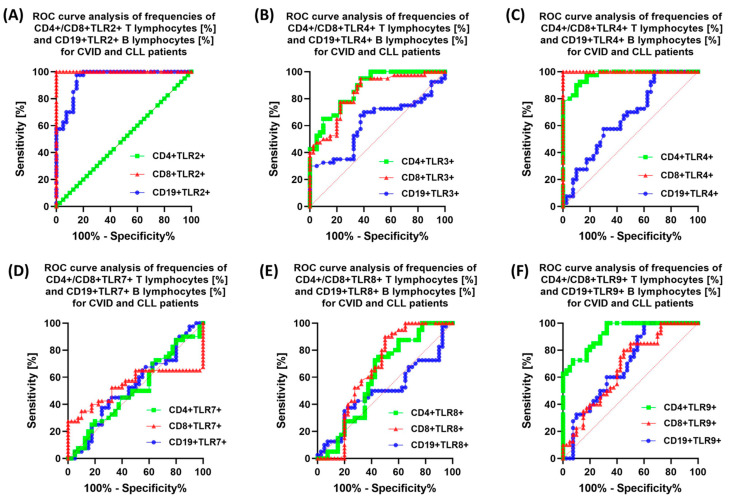
ROC curves for the assessment of the percentage of analyzed T and B lymphocyte subpopulations showing positive expression of the tested TLRs between patients diagnosed with CVID and CLL. (**A**) ROC curve for the percentage of analyzed T and B lymphocyte subpopulations expressing positive TLR2; (**B**) ROC curve for the percentage of analyzed T and B lymphocyte subpopulations expressing positive TLR3; (**C**) ROC curve for the percentage of analyzed T and B lymphocyte subpopulations expressing positive TLR4; (**D**) ROC curve for the percentage of analyzed T and B lymphocyte subpopulations expressing positive TLR7; (**E**) ROC curve for the percentage of analyzed T and B lymphocyte subpopulations expressing positive TLR8; (**F**) ROC curve for the percentage of analyzed T and B lymphocyte subpopulations expressing positive TLR9.

**Figure 14:**
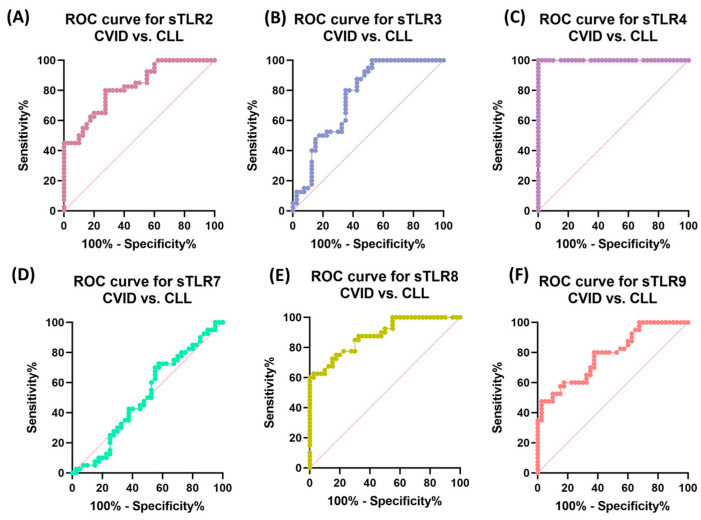
ROC curves for the assessment of the serum concentration of tested sTLRs between patients diagnosed with CVID and CLL. (**A**) ROC curve for the serum concentration of sTLR2; (**B**) ROC curve for the serum concentration of sTLR3; (**C**) ROC curve for the serum concentration of sTLR4; (**D**) ROC curve for the serum concentration of sTLR7; (**E**) ROC curve for the serum concentration of sTLR8; (**F**) ROC curve for the serum concentration of sTLR9.

Due to the observed differences in the expression profiles of the tested TLRs on selected subpopulations of lymphocytes, as well as significant changes in their serum concentrations in immunodeficient patients compared to healthy volunteers, we decided to investigate whether they can become a kind of non-invasive biomarker of the development of these diseases. To this end, we performed a receiver operating characteristic (ROC) analysis for individual immunological parameters for patients with CVID and CLL in relation to healthy individuals, as well as between both types of immunodeficiencies. In the case of immunophenotype analysis, the highest sensitivity relative to control patients was obtained for patients with CVID for CD4+TLR2, CD8+TLR2+, CD8+TLR4+, and for TLR9 receptor expression for all lymphocyte subpopulations tested (Supplementary Materials—Table S1). Similar results were obtained for CLL patients, for whom the highest sensitivity was observed for: CD8+TLR2+, CD19+TLR2+, TLR4, and TLR9 expression for all lymphocyte subpopulations tested (Supplementary Materials—Table S1). Changes in the serum concentration of all tested TLRs were also diagnostically significant in the case of both types of deficiencies compared to healthy individuals (Supplementary Materials—Table S1). Due to the relationships found in both subtypes of the analyzed immunodeficiencies, we conducted a ROC analysis aimed at indicating which of the analyzed parameters could become a potential biomarker. The obtained data are shown in Table 7 and Table 8 and Figure 13A–F and Figure 14A–F.

When evaluating the analyzed subpopulations of lymphocytes positive for the tested TLRs between CVID and CLL patients, the following have the greatest potential: CD4+TLR2+, CD8+TLR2+, CD19+TLR2+, as well as CD8+TLR4+ and CD4+TLR9+ (Table 8, Figure 13A,B,F).

Serum concentrations of almost all tested sTLRs were also highly sensitive and specific, with the exception of sTLR7 (Table 8) (Figure 14A–F). However, the concentration of sTLR4 showed the greatest potential (Table 8) (Figure 14C).

## 4. Discussion

Dysfunction of TLR signaling in immunodeficiencies refers to abnormalities or impairments in the functioning of these receptors, which are key components of the innate immune system. TLRs play a significant role in recognizing and responding to various pathogens, triggering immune responses essential to fighting infection. However, there are defects or deficiencies in the TLR signaling pathways in immunodeficiencies, leading to an impaired immune response [13,33,34]. Deregulation of TLR signaling can manifest itself in a variety of ways. It may involve genetic mutations or changes in genes encoding TLRs or downstream signaling molecules. These genetic abnormalities can interfere with the proper functioning of the TLRs, resulting in reduced or no response to pathogens. Consequently, immunodeficient individuals may experience increased susceptibility to infection and impaired ability to effectively eliminate pathogens [35,36,37].

In addition, TLR signaling dysfunction can lead to immune response dysregulation. In some cases, TLR signaling pathways may be exaggerated or overactivated, resulting in chronic inflammation or autoimmune symptoms. On the other hand, some immunodeficiencies may show reduced TLR signaling, leading to an inappropriate immune response against pathogens [38,39,40].

The dysfunction of TLR signaling in immunodeficiencies highlights TLRs’ critical role in maintaining a functional immune system. Understanding and addressing these dysfunctions may contribute to developing targeted therapies and interventions for immunodeficient individuals to restore or enhance TLR-mediated immune responses [10,13,33]. However, there is a lack of comprehensive studies to explain disorders of TLR signaling pathways in the course of individual disease subtypes of both PID and SID. In our research, we presented two diseases classified as PIDs and SIDs, in which signaling pathways are dysregulated with the use of TLR, which may contribute to the immunopathogenesis of these diseases.

Despite the prevalence of CVID, studies on changes in TLR expression levels are scarce. Abnormalities in Toll-like receptor signaling have been observed in some patients with CVID. The analysis of data from PubMed or Web of Science in terms of the number of publications on the role of TLR in the course of PID and SID showed only several dozen literature items in recent years relating to this important issue. In the case of PID, we found seventy-three articles, of which only three directly related to the disease entity we studied, i.e., CVID. The first, published in 2009 by Yu et al. [41], concerned the importance of TLR7, TLR8, and TLR9 in the context of the functioning of B lymphocytes and pDCs. Their research focused on the process of proliferation, isotype switching, and immunoglobulin production by B lymphocytes under controlled and diseased conditions (with particular emphasis on naive and memory B cell subsets mediated by the TLRs mentioned above). Their study results showed that, unlike control CD27+ B cells, CVID B cells activated with TLR7, TLR7/8, or TLR9, as well as isolated CD27+ B cells, did not proliferate, upregulate CD27, or shed surface IgD. TLR-stimulated CVID B cells also failed to increase activation-induced cytosine deaminase mRNA or produce IgG and IgA. Additionally, TLR7-stimulated PBMCs and pDCs showed minimal or no IFN-α production. However, when IFN-α was reconstituted in TLR7-stimulated CVID B cell cultures, it facilitated proliferation, CD27 upregulation, and isotype switching. The authors emphasize that these TLR defects are specific to CVID, as PBMCs from CVID patients stimulated with TLR ligands produced normal amounts of TNF-α, IL-6, and IL-12, and TLR3-mediated expression of IFN-β by CVID fibroblasts was also normal. Although the presented study results show a slightly different aspect of the role of TLRs in the course of CVID than those presented in our publication, they draw important attention to the fact that the TLR7 and TLR9 signaling pathways are clearly defective in CVID, suggesting that one or more effector molecules common to these related activation pathways may be abnormal. This is also emphasized by our research, in which we not only showed abnormalities in the percentage of TLR7, TLR8, and TLR9 occurrence on individual subpopulations of lymphocytes but also showed the existence of correlations between the individual TLRs studied.

The results of this study published by Yu et al. found their continuation in the 2014 paper by Taraldsrud et al. [42] in which researchers showed that patients with CVID have a lower number and frequency of DCs in the blood and there is evidence of impaired TLR activation, and their observations suggest a possible primary disadvantage in generating functional DCs. The published study results revealed that patients with CVID had decreased absolute numbers of both DC subsets in their blood. However, this decrease in numbers was not reflected in the reduced frequencies of CD34(+) pDC precursors in the bone marrow. Moreover, at the single-cell level, DCs from CVID patients and healthy controls produced similar amounts of IFN-α and IL-12 and expressed similar levels of activation markers in response to human cytomegalovirus and ligands for TLR7 and TLR9. The authors emphasize that their study represents the most comprehensive functional characterization of naturally occurring DCs in CVID to date and is the first to evaluate bone marrow progenitor cell production. Moreover, they also suggest that, based on these findings, it seems unlikely that CVID is the result of insufficient production of naturally occurring DCs or a defect in their signaling by TLR7 or TLR9.

Research conducted by Trujillo’s team in 2011 [43] assessed the frequency and functional response of innate immune cells in peripheral blood from CVID patients and healthy controls after activation with TLR2 agonists, TLR4, and TLR9. Their study showed that the frequency of CD1d-restricted invariant T-NK T cells was significantly reduced in patients with CVID. There was also a noticeable, though not statistically significant, decrease in the absolute numbers of plasmacytoid dendritic cells and natural killer cells in these patients. The authors also emphasize that after stimulation with TLR ligands, CD80 and CD86 expression on innate cells was not changed in patients with CVID, although three patients showed low baseline levels of these surface molecules on monocytes compared to healthy controls. Additionally, the study authors observed a significant increase in TNF-α levels in mononuclear cell supernatants from CVID patients after lipopolysaccharide stimulation. These findings led the authors to draw attention to the abnormalities of the innate immune response in some patients with CVID, which, according to the researchers, opens up the possibility of evaluating innate immunity genes as potential candidates to explain the clinical phenotype of CVID.

Another paper that addresses this issue is an article by Marron et al. of 2012 [44], which is an extensive review of the TLR function in primary B cells. The authors cite the previously reported research by Yu et al. but also highlight that TLR dysregulation and/or abnormalities may be associated with the development of autoimmunity. This is due to the presence of microbial DNA and RNA patterns that overlap to some extent with human DNA and RNA, and host nucleotide fragments may inadvertently induce autoimmune reactions. In support of this thesis, the authors cite a case study of a patient with SLE who showed defects in TLR7 and TLR9, which later developed into CVID, resulting in regression of clinical lupus. This suggests that previously intact TLR7 and TLR9 signaling may have contributed to the development of autoimmunity [45]. Other studies by the Chapel team [46] and Cunningham-Rundles [47] showed that despite reduced IFN-α production by TLR-activated CVID plasmacytoid dendritic cells (pDCs), approximately 20% of patients still experience autoimmune complications, with immune thrombocytopenic purpura and hemolytic anemia as the most common symptoms. Potential explanations for this, according to the authors, include TLR stimulation of a predominantly naive B cell population containing self-reactive cells or aberrant TLR signaling that interferes with the regulation of other activation pathways. Notably, patients with mutations in MyD88, UNC-93B, or IRAK4 show an increased number of circulating autoreactive B cells in the periphery. However, these patients do not have elevated levels of autoantibodies in their blood, nor do they have an increased number of autoimmune diseases.

All of the studies reported show altered expression or function of TLRs in certain subgroups of immune cells, such as monocytes or dendritic cells, in a subset of patients with CVID. These changes in Toll-like receptor signaling can potentially contribute to the immune system’s dysregulation and increased susceptibility to infection seen in CVID [22,41,48,49]. This is also shown by our research, which showed an increased percentage of TCD4+, TCD8+, and CD19+ lymphocytes positively expressing TLR, TLR3, TLR4, TLR7, TLR8, or TLR9, as well as the concentration of their soluble forms in the serum. It should be noted that the relationship between Toll-like receptors and CVID is still being explored, and more research is needed to fully understand their interplay. The studies presented by us are only a fragment of the changes that occur in the immune system in the course of CVID, which is a complex disorder with various abnormalities, not only immunological but also genetic, and the role of Toll-like receptors in the context of CVID is not yet fully explained.

The analysis of the available literature on the role of TLRs in the course of SID, and in particular the one studied by our CLL team, showed the availability of 56 articles, of which only a dozen referred directly to the assessment of the function of individual TLRs. The vast majority of articles focus on assessing the function of TLRs (especially TLR9) on B lymphocytes and the effectiveness of various therapeutic agents. Work by Mongini et al. from 2015 [50] and Gupta et al. from 2018 [51] highlights the synergy of TLR9 and IL-15 that promote in vitro clonal expansion of CLL-B cells. Another paper on TLR9 is Dampmann et al. of 2020 [52], which examined the effects of Toll-like receptor 9 (TLR9) agonists, CpG types A, B, and C, as well as oligodeoxynucleotide (ODN) antagonist INH-18, on cell polarization and migration of primary human CLL cells. The investigators showed that, along with an increased frequency of morphologically polarized cells, CLL cells stimulated with type B and C CpG showed higher migratory activity in vitro and, when injected intravenously, showed a higher frequency of homing to the bone marrow of NOD.Cg-Prkdc^scid^ Il2rg^tm1Wjl^/SzJ (NSG) mice. These findings suggest that, independently of TLR9 signaling, B-type and C-type CpGs promote the process of cell polarization of CLL cells and increase their migratory capacity both in vitro and *in vivo* [52]. The research presented by Wagner et al. of 2016 [53] in which the authors discussed heterogeneous responses to TLR agonists is related to differences in the ability of CLL cells to activate BCR-related Syk kinase. The researchers indicate that ZAP-70 expression is critical for TLR9-mediated Syk activation and that Syk activation provides an anti-apoptotic signal independent of Mcl-1, Bcl-2, and Bcl-XL but associated with the degradation of pro-apoptotic Bim. Mechanistically, TLR9-mediated anti-apoptotic signals in ZAP-70-positive CLL cells lead to the secretion of immunoglobulin M, which serves as the (auto)antigen that triggers the pro-survival BCR signal. Studies also indicate that the integration of TLR signaling with the adaptive immune response may further promote CLL cell survival and may contribute to the poor prognosis of ZAP-70-positive CLL. This study sheds light on the complex interactions between TLR9 and BCR signaling pathways and their impact on CLL cell survival, potentially guiding the development of targeted therapies for CLL patients with specific genetic markers [53].

Research conducted by the Muzio team in 2009 [54] showed that CLL cells express various pattern recognition receptors, including TLR1, TLR2, TLR6, TLR10, NOD1, and NOD2. It was also found that when stimulated with TLR1/2/6 ligands such as bacterial lipopeptides, leukemic cells activated the nuclear factor-kappaB signaling pathway and expressed CD86 and CD25 activating molecules, leading to protection against spontaneous apoptosis. The authors stress that these findings provide further evidence to support the idea that CLL cells resemble antigen-activated B cells. In addition, they suggest that TLRs may play a potential role in modulating the response of CLL cells in the context of specific antigen recognition [54]. The results of these studies are identical to the results obtained by our team, in which the percentage of B lymphocytes, as well as TCD4+ and CD8+ lymphocytes, positively expressing TLR2-TLR4 and TLR7-TL9 was higher in patients with CLL compared to healthy volunteers included in the study.

Similar research results were obtained by our colleagues from the Department of Clinical Immunology [55], who in their work assessed the role of TLR2 on B cells in patients with CLL. Their study showed that CLL patients with poor prognostic factors, such as ZAP-70 and/or CD38 expression as well as 17p and/or 11q deletion, had a lower percentage of TLR2+/CD19+ cells. On the other hand, among patients with del(13q14) associated with a favorable prognosis, the percentage of TLR2+/CD19+ cells was higher than in patients with del(11q22) and/or del(17p13) and even higher than in the control group. Moreover, the researchers emphasize that the low percentage of CD19+/CD5+/TLR2+ cells was associated with shorter treatment duration. In addition, a low percentage of CD19+/CD5+ TLR2-positive cells was associated with overall survival (OS) in patients with CLL. Patients with a percentage of 1.6% or more TLR2-positive CD5+ B cells, as determined by receiver performance curve analysis, had a longer time to treatment and longer OS compared to a group with a lower percentage of TLR2-positive cells. The researchers have suggested that the expression level of TLR2 on leukemic B cells may be an important contributor to the immune dysfunction in CLL patients [55].

Another study by Daniela Rozková and colleagues in 2010 [56] showed that B-CLL cells express a similar set of TLRs as memory B cells from healthy donors, including TLR 1, TLR2, TLR6, TLR7, and TLR9. However, B-CLL cells lacked expression of TLR4, unlike memory B cells. TLR expression correlated with the ability of cells to respond to specific TLR agonists, with ODN2006 (TLR9 agonist) being the strongest stimulus at the phenotypic level. B-CLL cells also responded to stimulation with loxoribine, Pam3CSK4, and MALP-2 (TLR7, TLR1/TLR2, and TLR2/TLR6 agonists, respectively). Stimulation of TLR7 and TLR9 induced IL-6 and TNF-alpha production. In 47% of the patients studied, treatment with ODN2006, MALP-2, and Pam3CSK4 reduced the survival of leukemic cells. In addition, the TLR9 agonists loxoribine, MALP-2, and Pam3CSK4 significantly induced proliferation of B-CLL cells. Interestingly, the authors emphasize that TLR stimulation led to the expression of CD38, a negative prognostic marker, on B-CLL cells. This expression can be induced directly by stimulation of TLR7 and TLR9 or indirectly by a soluble factor produced in cells other than B-CLL after stimulation with TLR2, TLR3, or TLR5 agonists [56].

A publication by Tan et al. from 2015 [57] indicates that a subset of B cell receptors (BCRs) of CLLs interact with antigens expressed on apoptotic cells, indicating that CLLs of BCRs can potentially internalize fragments containing RNA or DNA from apoptotic cells, leading to activation of TLR7 or TLR9, respectively. cAMP phosphodiesterase type 4 (PDE4) inhibitors, by blocking cAMP degradation, activate cAMP-mediated signaling and induce apoptosis in CLL cells. In this study, it was shown that irradiated leukemic cells from the same patient trigger proliferation in CLL cells, and this proliferation can be inhibited by the TLR7/8/9 inhibitor, DNase, and the PDE4 inhibitor, rolipram. These findings suggest that PDE4 inhibitors may have clinical potential in the treatment of CLL or autoimmune diseases driven by TLR-mediated signaling [57].

A study presented by the Martínez-Trillos team in 2014 [58] evaluated the occurrence and characteristics of mutations in the TLR2, TLR5, and TLR6 receptor genes and myeloid differentiation of the primary response 88 (MYD88) in patients with CLL. Of the 587 CLL patients analyzed, 3.9% were mutated, of which 19 were MYD88 (1 patient also had an IRAK1 co-mutation), 2 were TLR2 (1 patient had a TLR6 co-mutation), 1 was IRAK1, and 1 was TLR5. No mutations were detected in IRAK2 and IRAK4. The authors also emphasize that cases of CLL patients with TLR/MYD88 mutations showed overexpression of genes related to the nuclear factor κB pathway and that patients with TLR/MYD88 mutations were significantly younger, 83% of them were ≤50 years of age, compared to patients without the mutation. Overall survival (OS) was significantly better in patients with the TLR/MYD88 mutation compared to non-mutated patients in the overall group as well as in the subgroup of patients ≤ 50 years of age (100% vs. 70%; *p* = 0.02). In addition, the study authors showed that the relative OS of patients with the TLR/MYD88 mutation was similar to that of the age- and sex-matched population [58].

Work by Dadashian et al. of 2019 [59] evaluated the activation of TLR signaling in lymph node-resident CLL cells and its partial inhibition by ibrutinib as indicated. Their research hypothesis was that ibrutinib only partially inhibits other signaling pathways that support CLL cell survival. The researchers noted that in normal B cells, TLR signaling works with B cell receptor (BCR) signaling to activate NF-κB. In their study, they showed that the experimentally confirmed TLR activation gene signature is increased in CLL cells located in lymph nodes compared to cells in the blood. In addition, we observed phosphorylation of NF-κB, STAT1, and STAT3 in lymph node-resident CLL cells and in cells stimulated with CpG oligonucleotides in vitro, confirming TLR activation. In addition, the researchers showed that CpG-induced TLR signaling was significantly hampered by both the IRAK1/4 inhibitor and ibrutinib. While the inhibition of TLR signaling was not complete with either drug alone, the combination of the two resulted in better results, particularly in more effective inhibition of TLR-mediated survival signaling. The obtained results suggested a significant role of TLR signaling in the pathogenesis of CLL and the maintenance of CLL cell survival during ibrutinib therapy [59].

Despite showing a slightly different part of the role of TLRs in the pathogenesis of CLL, these studies are consistent with the results obtained by us, in which we confirm the deregulation of TLR-based signaling pathways. Studies have shown that CLL cells can express various Toll-like receptors, including TLR1, TLR2, TLR4, TLR7, and TLR9. These receptors can be found on the surface of CLL cells or within specialized compartments inside the cells [60,61,62]. Activation of Toll-like receptors triggers signaling pathways that lead to the production of inflammatory molecules and activation of immune responses. In CLL, the activation of specific Toll-like receptors, such as TLR9, has been shown to promote CLL cell survival and proliferation [59,61,62]. Genetic variations or polymorphisms in Toll-like receptors have been investigated for their potential association with CLL susceptibility, disease progression, and treatment response. However, the results have been inconsistent, and more research is needed to establish clear associations [63,64,65,66]. Our analyses emphasize the importance of dysregulation of TLR-based signaling pathways in the course of CLL. It should be noted that the role of TLRs in CLL is still an active area of research, and the exact impact and clinical implications are not fully understood. The relationship between CLL and TLR is complex, and more research is needed to better characterize their interaction and potential therapeutic applications. However, given their significant role in the immunopathogenesis of CLL, there may be interest in developing therapies that modulate TLR signaling in the future. For example, TLR agonists have been studied as potential therapeutic agents for enhancing immune responses against CLL cells.

## 5. Conclusions

In immunodeficiencies, the state of the immune system may vary depending on the specific type of immunodeficiency and its causes. In PIDs, which are often genetic, the immune system may be affected from birth or early childhood. Deficiencies in specific components of the immune system, such as T cells, B cells, or antibodies, typically characterize these conditions. As a result, people with PIDs can exhibit varying degrees of immune system dysfunction, from mild to severe. On the other hand, SIDs can develop later in life due to factors such as infections, medications, underlying medical conditions, or treatment such as chemotherapy. In these cases, the immune system may initially function normally but become weakened due to specific triggers or ongoing factors. The state of the immune system in SIDs can vary depending on the cause and duration of the deficiency. It should be noted that in some cases, SIDs may be reversible if the underlying cause is identified and treated successfully. Understanding the intricate relationship between TLR and PIDs and SIDs is critical to understanding the underlying mechanisms of the disease and developing targeted therapeutic strategies. We hope that the research results presented in this article contribute not only to the deepening of knowledge on this important topic but also serve as an inspiration for other scientists and clinicians to develop and implement new therapies for treating immunodeficiencies based on TLR signaling pathways.

## Figures and Tables

**Table 1:** Selected Parameters of Morphology and Concentration of Peripheral Blood Immunoglobulins.

Parameter	Patients with CVID (n = 40)		Patients with CLL (n = 40)		Healthy Volunteers (n = 40)		*p*-Value	*p*-Value		
	Mean ± SD	Median (Range)	Mean ± SD	Median (Range)	Mean ± SD	Median (Range)		CVID Vs. CLL	CVID Vs. Healthy Volunteers	CLL Vs. Healthy Volunteers
WBC	6.92 ± 1.63	6.70 (4.78–9.99)	31.95 ± 11.35	34.19 (10.34–47.85)	5.66 ± 1.39	6.03 (3.40–7.78)	0.000 *	0.000 *	0.004 *	0.000 *
LYM	0.96 ± 0.54	0.92 (0.08–1.99)	22.16 ± 5.21	21.26 (5.54–35.60)	1.56 ± 0.49	1.49 (0.76–2.85)	0.000 *	0.000 *	0.000 *	0.000 *
MON	0.47 ± 0.15	0.47 (0.20–0.79)	0.57 ± 0.45	0.42 (0.09–1.71)	0.48 ± 0.15	0.48 (0.20–0.81)	0.733	0.642	0.556	0.524
NEU	1.0 ± 0.56	1.14 (0.03–1.92)	3.31 ± 1.44	3.15 (0.61– 7.40)	3.35 ± 1.27	3.50 (0.91–5.52)	0.000 *	0.000 *	0.000 *	0.629
EOS	0.08 ± 0.03	0.08 (0.03–0.19)	0.12 ± 0.10	0.10 (0.01–0.51)	0.10 ± 0.04	0.09 (0.03–0.22)	0.182	0.200	0.058	0.698
BAS	0.03 ± 0.02	0.03 (0.00–0.08)	0.08 ± 0.05	0.07 (0.01–0.21)	0.04 ± 0.02	0.03 (0.00–0.09)	0.000 *	0.000 *	0.103	0.000 *
RBC	3.39 ± 0.24	3.39 (2.86–3.89)	4.21 ± 0.70	4.18 (2.34–5.21)	4.51 ± 0.32	4.53 (3.81–5.19)	0.000 *	0.000 *	0.000 *	0.038 *
HGB	9.98 ± 0.67	9.98 (8.33–11.63)	12.27 ± 1.71	12.50 (8.20–14.40)	13.30 ± 0.90	13.30 (11.10–15.50)	0.000 *	0.000 *	0.000 *	0.004 *
PLT	115.43 ± 18.24	114.09 (86.36–148.42)	122.85 ± 41.52	116.00 (34.00–198.00)	244.50 ± 64.11	233.50 (152.00–404.00)	0.000 *	0.298	0.000 *	0.038 *
IgG	5.08 ± 0.86	5.19 (2.96–6.58)	5.37 ± 3.51	5.32 (0.31–12.93)	12.40 ± 2.11	12.65 (7.23–16.06)	0.000 *	0.785	0.000 *	0.038 *
IgM	1.37 ± 0.53	1.38 (0.37–2.38)	1.79 ± 1.10	1.44 (0.23–4.37)	1.47 ± 0.57	1.49 (0.40–2.56)	0.000 *	0.000 *	0.370	0.038 *
IgA	0.62 ± 0.23	0.64 (0.11–1.09)	0.79 ± 0.58	0.52 (0.05–3.38)	2.07 ± 0.76	1.95 (0.70–4.00)	0.000 *	0.000 *	0.000 *	0.049 *

* Statistically significant results.

**Table 2:** Comparative Analysis of Peripheral Blood Immunophenotype in Patients with CVID and CLL Compared to Healthy Volunteers.

Frequency of Individual Populations of Immune System Cells [%]	Patients with CVID (n = 40)		Patients with CLL (n = 40)		Healthy Volunteers (n = 40)		*p*-Value	*p*-Value		
	Mean ± SD	Median (Range)	Mean ± SD	Median (Range)	Mean ± SD	Median (Range)		CVID Vs. CLL	CVID Vs. Healthy Volunteers	CLL Vs. Healthy Volunteers
CD45+ leukocytes [%]	90.72 ± 4.60	91.04 (79.38–98.91)	96.42 ± 2.30	97.29 (88.75–99.12)	95.28 ± 2.75	95.88 (89.14–99.37)	0.000 *	0.000 *	0.000 *	0.043 *
CD3+ T lymphocytes [%]	66.56 ± 9.67	65.51 (44.85–89.85)	21.17 ± 10.52	16.82 (5.2–41.12)	72.83 ± 6.31	71.94 (65.09–96.34)	0.000 *	0.000 *	0.001*	0.000 *
CD19+ B lymphocytes [%]	8.33 ± 4.71	7.14 (1.45–20.94)	73.17 ± 12.96	79.04 (46.21–91.91)	12.59 ± 2.25	12.46 (7.81–16.83)	0.000 *	0.000 *	0.000 *	0.000 *
NK cells [%]	15.84 ± 6.86	14.37 (3.34–28.70)	2.79 ± 0.96	2.94 (0.98–4.17)	10.18 ± 3.03	8.55 (7.13–16.43)	0.000 *	0.000 *	0.000 *	0.000 *
CD4+CD3+ T lymphocytes [%]	30.77 ± 11.05	28.62 (10.45–56.73)	13.51 ± 7.09	10.34 (3.24–26.84)	47.53 ± 4.76	46.76 (42.19–65.51)	0.000 *	0.000 *	0.000 *	0.000 *
CD8+CD3+ T lymphocytes [%]	35.78 ± 12.90	35.40 (15.10–58.76)	7.67 ± 3.84	6.06 (2.08–15.21)	26.39 ± 2.98	26.91 (20.18–31.07)	0.000 *	0.000 *	0.001 *	0.000 *
CD4+CD3+/CD8+CD3+ ratio	1.09 ± 0.77	0.75 (0.22–3.47)	1.81 ± 0.43	1.78 (0.52–3.47)	1.82 ± 0.25	1.78 (1.53–2.23)	0.000 *	0.000 *	0.000 *	0.882

* Statistically significant results.

**Table 3:** Investigation of the Percentage of Selected Subpopulations of Peripheral Blood Lymphocytes Showing Positive Expression of Tested TLRs.

Parameter		Patients with CVID (n = 40)		Patients with CLL (n = 40)		Healthy Volunteers (n = 40)		*p*-Value	*p*-Value		
		Mean ± SD	Median (Range)	Mean ± SD	Median (Range)	Mean ± SD	Median (Range)		CVID Vs. CLL	CVID Vs. Healthy Volunteers	CLL Vs. Healthy Volunteers
TLR2	CD4+TLR2+ T lymphocytes [%]	6.02 ± 0.56	5.99 (5.04–6.96)	2.10 ± 0.70	2.14 (0.63–3.89)	0.64 ± 0.40	0.76 (0.02–1.45)	0.000 *	0.000 *	0.000 *	0.000 *
	CD8+TLR2+ T lymphocytes [%]	6.63 ± 0.88	6.87 (5.00–7.80)	2.95 ± 0.56	2.95 (2.03–3.86)	0.96 ± 0.60	1.09 (0.04–1.88)	0.000 *	0.000 *	0.000 *	0.000 *
	CD19+TLR2+ B lymphocytes [%]	4.80 ± 1.06	4.64 (3.01–6.76)	2.85 ± 0.53	2.82 (2.03–3.78)	0.60 ± 0.30	0.67 (0.14–1.29)	0.000 *	0.000 *	0.000 *	0.000 *
TLR3	CD4+TLR3+ T lymphocytes [%]	3.40 ± 0.87	3.50 (2.02–4.91)	2.11 ± 0.60	2.13 (0.60–3.16)	0.94 ± 0.59	1.06 (0.03–2.13)	0.000 *	0.000 *	0.000 *	0.000 *
	CD8+TLR3+ T lymphocytes [%]	2.99 ± 0.55	3.04 (2.02–3.98)	1.89 ± 0.95	2.22 (0.29–3.60)	0.79 ± 0.51	0.63 (0.12–1.83)	0.000 *	0.000 *	0.000 *	0.000 *
	CD19+TLR3+ B lymphocytes [%]	3.03 ± 0.64	3.19 (2.12–3.98)	2.64 ± 0.95	2.59 (1.01–4.41)	0.49 ± 0.25	0.52 (0.12–1.12)	0.000 *	0.07	0.000 *	0.000 *
TLR4	CD4+TLR4+ T lymphocytes [%]	6.70 ± 0.87	6.78 (5.21–7.97)	4.34 ± 0.85	4.15 (3.19–5.97)	0.98 ± 0.45	1.07 (0.14–1.76)	0.000 *	0.000 *	0.000 *	0.000 *
	CD8+TLR4+ T lymphocytes [%]	6.40 ± 0.81	6.32 (5.00–7.92)	4.12 ± 0.55	4.19 (3.01–4.97)	0.98 ± 0.61	1.07 (0.01– 1.79)	0.000 *	0.000 *	0.000 *	0.000 *
	CD19+TLR4+ B lymphocytes [%]	4.60 ± 0.98	4.74 (3.04–5.98)	3.92 ± 1.21	3.96 (2.00–5.95)	0.74 ± 0.35	0.79 (0.17–1.60)	0.000 *	0.012 *	0.000 *	0.000 *
TLR7	CD4+TLR7+ T lymphocytes [%]	1.99 ± 0.52	2.00 (1.03–2.97)	1.98 ± 0.51	2.01 (1.04–2.97)	0.49 ± 0.30	0.55 (0.02–1.20)	0.000 *	0,889	0.000 *	0.000 *
	CD8+TLR7+ T lymphocytes [%]	2.06 ± 0.53	2.09 (1.06–2.92)	2.12 ± 1.22	2.40 (0.26–4.47)	0.51 ± 0.38	0.34 (0.02–1.31)	0.000 *	0.550	0.000 *	0.000 *
	CD19+TLR7+ B lymphocytes [%]	2.89 ± 0.59	2.82 (2.00–3.97)	2.94 ± 0.56	2.88 (2.12–3.90)	0.43 ± 0.18	0.44 (0.08–0.74)	0.000 *	0.615	0.000 *	0.000 *
TLR8	CD4+TLR8+ T lymphocytes [%]	2.89 ± 1.21	3.26 (0.78–4.93)	2.49 ± 0.89	2.52 (1.08–3.93)	0.79 ± 0.51	0.76 (0.09–1.77)	0.000 *	0.093	0.000 *	0.000 *
	CD8+TLR8+ T lymphocytes [%]	2.44 ± 1.17	2.54 (0.14–4.08)	1.94 ± 0.54	1.82 (1.05–3.00)	0.42 ± 0.27	0.33 (0.06–0.93)	0.000 *	0.014 *	0.000 *	0.000 *
	CD19+TLR8+ B lymphocytes [%]	2.43 ± 0.84	2.41 (1.02–3.96)	2.43 ± 0.99	2.39 (1.05–4.28)	0.56 ± 0.28	0.59 (0.14–1.33)	0.000 *	0.996	0.000 *	0.000 *
TLR9	CD4+TLR9+ T lymphocytes [%]	5.03 ± 0.58	4.96 (4.15–5.95)	3.76 ± 0.69	3.81 (2.07–4.78)	0.55 ± 0.35	0.54 (0.07–1.22)	0.000 *	0.000 *	0.000 *	0.000 *
	CD8+TLR9+ T lymphocytes [%]	4.44 ± 0.75	4.63 (3.14–5.88)	3.98 ± 0.60	3.98 (3.02–4.94)	0.69 ± 0.45	0.64 (0.08–1.61)	0.000 *	0.006 *	0.000 *	0.000 *
	CD19+TLR9+ B lymphocytes [%]	4.61 ± 0.87	4.56 (3.03–5.98)	4.06 ± 0.58	4.07 (3.09–4.94)	0.78 ± 0.37	0.84 (0.18–1.65)	0.000 *	0.003 *	0.000 *	0.000 *

* Statistically significant results.

**Table 4:** The concentration of soluble forms of TLRs in the serum of immunodeficient patients in relation to healthy volunteers.

Serum Concentration [ng/mL]	Patients with CVID (n = 40)		Patients with CLL (n = 40)		Healthy Volunteers (n = 40)		*p*-Value	*p*-Value		
	Mean ± SD	Median (Range)	Mean ± SD	Median (Range)	Mean ± SD	Median (Range)		CVID Vs. CLL	CVID Vs. Healthy Volunteers	CLL Vs. Healthy Volunteers
sTLR2	13.83 ± 2.31	13.80 (10.11–17.84)	10.66 ± 2.34	10.61 (7.18–14.67)	1.89 ± 0.66	1.95 (1.00–2.93)	0.000 *	0.000 *	0.000 *	0.000 *
sTLR3	9.00 ± 1.34	8.87 (7.10–11.85)	7.87 ± 0.56	7.83 (7.04–8.97)	1.57 ± 0.81	1.47 (0.04–2.97)	0.000 *	0.000 *	0.000 *	0.000 *
sTLR4	15.52 ± 1.33	15.55 (13.31–17.89)	11.08 ± 1.09	11.09 (9.01–12.85)	3.13 ± 0.55	3.29 (2.12–3.94)	0.000 *	0.000 *	0.000 *	0.000 *
sTLR7	6.58 ± 0.90	6.56 (5.10–7.98)	6.56 ± 0.78	6.61 (5.17–7.87)	1.07 ± 0.59	1.07 (0.06–1.93)	0.000 *	0.859	0.000 *	0.000 *
sTLR8	8.69 ± 0.92	8.69 (7.13–9.99)	6.81 ± 1.14	6.72 (5.17–8.94)	0.97 ± 0.63	1.01 (0.01–1.98)	0.000 *	0.000 *	0.000 *	0.000 *
sTLR9	13.06 ± 1.05	12.89 (11.18–14.83)	11.54 ± 1.38	11.67 (9.18–13.68)	3.00 ± 0.58	2.96 (2.00–3.92)	0.000 *	0.000 *	0.000 *	0.000 *

* Statistically significant results.

**Table 5:** Statistically significant correlations of selected immunological parameters for patients diagnosed with CVID.

Parameters	R Spearman	t(N-2)	*p*-Value
Percentage of occurrence CD19+TLR2+ and CD8+TLR3+	−0.364	−2.47	0.017 *
Percentage of occurrence CD19+TLR4+ and CD4+TLR8+	−0.319	−2.13	0.039 *
Percentage of occurrence CD19+TLR3+ and CD19+TLR8+	0.320	2.14	0.038 *
Percentage of occurrence CD19+TLR3+ and CD4+TLR9+	0.360	2.44	0.019 *
Percentage of occurrence CD4+TLR4+ and CD4+TLR7+	−0.356	−2.40	0.020 *
Percentage of occurrence CD4+TLR4+ and CD8+TLR4+	0.366	2.48	0.017 *
Percentage of occurrence CD4+TLR4+ and CD8+TLR3+	0.322	2.15	0.037 *
Percentage of occurrence CD4+TLR4+ and CD8+TLR9+	0.345	2.32	0.025 *
Percentage of occurrence CD4+TLR8+ and CD8+TLR4+	−0.542	−4.08	0.0002 *
Percentage of occurrence CD4+TLR9+ and CD8+TLR3+	−0.306	−2.03	0.048 *
Percentage of occurrence CD4+TLR9+ and CD8+TLR7+	−0.327	−2.18	0.034 *
Percentage of occurrence CD19+TLR4+ and serum concentration sTLR9	−0.304	−2.02	0.049 *
Percentage of occurrence CD19+TLR8+ and serum concentration sTLR7	−0.313	−2.08	0.043 *
Percentage of occurrence CD4+TLR2+ and serum concentration sTLR2	0.308	2.05	0.046 *
Percentage of occurrence CD4+TLR7+ and serum concentration sTLR7	−0.313	−2.08	0.043 *
Percentage of occurrence CD8+TLR3+ and serum concentration sTLR9	0.356	2.41	0.020 *
Percentage of occurrence CD8+TLR7+ and serum concentration sTLR4	−0.405	−2.80	0.007 *
Serum concentration sTLR2 and serum concentration sTLR9	0.437	3.05	0.003 *

* Statistically significant results.

**Table 6:** Statistically significant correlations of selected immunological parameters for patients diagnosed with CLL.

Parameters	R Spearman	t(N-2)	*p*-Value
Percentage of occurrence CD19+TLR2+ and CD8+TLR4+	−0.370	−2.46	0.018 *
Percentage of occurrence CD19+TLR4+ and CD4+TLR4+	0.429	2.93	0.005 *
Percentage of occurrence CD19+TLR3+ and CD19+TLR8+	0.469	3.27	0.002 *
Percentage of occurrence CD19+TLR3+ and CD4+TLR2+	0.549	4.04	0.0002 *
Percentage of occurrence CD19+TLR3+ and CD4+TLR3+	0.620	4.88	0.000 *
Percentage of occurrence CD19+TLR3+ and CD4+TLR8+	0.608	4.72	0.000 *
Percentage of occurrence CD19+TLR3+ and CD4+TLR9+	0.403	2.72	0.009 *
Percentage of occurrence CD19+TLR3+ and CD8+TLR3+	0.605	4.68	0.000 *
Percentage of occurrence CD19+TLR3+ and CD8+TLR7+	0.615	4.81	0.000 *
Percentage of occurrence CD19+TLR3+ and CD8+TLR8+	0.622	4.90	0.000 *
Percentage of occurrence CD19+TLR3+ and CD8+TLR9+	0.350	2.30	0.026 *
Percentage of occurrence CD19+TLR8+ and CD4+TLR2+	0.350	2.30	0.026 *
Percentage of occurrence CD19+TLR8+ and CD4+TLR3+	0.404	2.72	0.009 *
Percentage of occurrence CD19+TLR8+ and CD4+TLR8+	0.555	4.11	0.000 *
Percentage of occurrence CD19+TLR8+ and CD8+TLR3+	0.656	5.37	0.000 *
Percentage of occurrence CD19+TLR8+ and CD8+TLR7+	0.570	4.28	0.000 *
Percentage of occurrence CD19+TLR8+ and CD8+TLR8+	0.549	4.05	0.0002 *
Percentage of occurrence CD4+TLR2+ and CD4+TLR3+	0.538	3.94	0.0003 *
Percentage of occurrence CD4+TLR2+ and CD4+TLR8+	0.579	4.38	0.000 *
Percentage of occurrence CD4+TLR2+ and CD4+TLR9+	0.403	2.71	0.009 *
Percentage of occurrence CD4+TLR2+ and CD8+TLR3+	0.632	5.03	0.000 *
Percentage of occurrence CD4+TLR2+ and CD8+TLR7+	0.573	4.31	0.0001 *
Percentage of occurrence CD4+TLR2+ and CD8+TLR8+	0.473	3.31	0.002 *
Percentage of occurrence CD4+TLR3+ and CD4+TLR8+	0.747	6.93	0.000 *
Percentage of occurrence CD4+TLR3+ and CD8+TLR3+	0.680	5.73	0.000 *
Percentage of occurrence CD4+TLR3+ and CD8+TLR7+	0.639	5.12	0.000 *
Percentage of occurrence CD4+TLR3+ and CD8+TLR8+	0.523	3.78	0.000 *
Percentage of occurrence CD4+TLR7+ and CD4+TLR9+	−0.461	−3.20	0.002 *
Percentage of occurrence CD4+TLR8+ and CD8+TLR3+	0.695	5.96	0.000 *
Percentage of occurrence CD4+TLR8+ and CD8+TLR7+	0.707	6.16	0.000 *
Percentage of occurrence CD4+TLR8+ and CD8+TLR8+	0.775	7.56	0.000 *
Percentage of occurrence CD4+TLR9+ and CD8+TLR3+	0.393	2.64	0.011 *
Percentage of occurrence CD8+TLR3+ and CD8+TLR7+	0.827	9.08	0.000 *
Percentage of occurrence CD8+TLR3+ and CD8+TLR8+	0.624	4.92	0.000 *
Percentage of occurrence CD8+TLR7+ and CD8+TLR8+	0.633	5.05	0.000 *
Percentage of occurrence CD19+TLR3+ and serum concentration sTLR8	−0.334	−2.18	0.034 *
Percentage of occurrence CD19+TLR9+ and serum concentration sTLR7	0.449	3.10	0.003 *
Percentage of occurrence CD4+TLR9+ and serum concentration sTLR8	−0.404	−2.72	0.009 *
Percentage of occurrence CD8+TLR2+ and serum concentration sTLR4	0.395	2.65	0.011 *
Percentage of occurrence CD8+TLR4+ and serum concentration sTLR8	−0.466	−3.25	0.002 *
Percentage of occurrence CD8+TLR8+ and serum concentration sTLR4	−0.328	−2.14	0.038 *

* Statistically significant results.

**Table 7:** ROC curve analysis for the percentage of occurrence of tested lymphocyte subpopulations showing positive expression of the analyzed TLRs.

Parameter	AUC	SE	+95%	−95%	Z Statistic	*p*-Value
CD4+/TLR2+ T lymphocytes [%]	1.00	0.00	1.00	1.00	-	0.000 *
CD8+TLR2+ T lymphocytes [%]	1.00	0.00	1.00	1.00	-	0.000 *
CD19+TLR2+ B lymphocytes [%]	0.95	0.022	0.906	0.993	20.201	0.000 *
CD4+/TLR3+ T lymphocytes [%]	0.874	0.037	0.802	0.947	10.119	0.000 *
CD8+TLR3+ T lymphocytes [%]	0.842	0.043	0.757	0.927	7.901	0.000 *
CD19+TLR3+ B lymphocytes [%]	0.617	0.065	0.491	0.744	1.815	0.0696
CD4+/TLR4+ T lymphocytes [%]	0.972	0.014	0.944	0.999	33.533	0.000 *
CD8+TLR4+ T lymphocytes [%]	1.00	0.00	1.00	1.00	-	0.000 *
CD19+TLR4+ B lymphocytes [%]	0.338	0.061	0.219	0.457	−2.667	0.007 *
CD4+/TLR7+ T lymphocytes [%]	0.509	0.065	0.382	0.637	0.144	0.885
CD8+TLR7+ T lymphocytes [%]	0.462	0.07	0.324	0.599	−0.548	0.583
CD19+TLR7+ B lymphocytes [%]	0.467	0.065	0.339	0.595	−0.508	0.611
CD4+/TLR8+ T lymphocytes [%]	0.391	0.065	0.263	0.518	−1.668	0.092
CD8+TLR8+ T lymphocytes [%]	0.499	0.066	0.37	0.629	−0.009	0.992
CD19+TLR8+ B lymphocytes [%]	0.341	0.065	0.214	0.468	-2.455	0.014 *
CD4+/TLR9+ T lymphocytes [%]	0.926	0.027	0.873	0.978	15.965	0.000 *
CD8+TLR9+ T lymphocytes [%]	0.678	0.06	0.560	0.795	2.957	0.003 *
CD19+TLR9+ B lymphocytes [%]	0.690	0.06	0.572	0.807	3.169	0.001 *

* Statistically significant results.

**Table 8:** ROC curve analysis for the serum concentration of tested sTLRs.

Serum concentration [ng/mL]	AUC	SE	+95%	−95%	Z Statistic	*p*-Value
sTLR2	0.822	0.045	0.733	0.910	7.138	0.000 *
sTLR3	0.756	0.055	0.647	0.864	4.624	0.000 *
sTLR4	1.000	0.000	1.000	1.000	-	0.000 *
sTLR7	0.512	0.066	0.383	0.640	0.176	0.860
sTLR8	0.881	0.036	0.810	0.952	10.501	0.000 *
sTLR9	0.785	0.050	0.687	0.882	5.707	0.000 *

* Statistically significant results.

## Data Availability

All information regarding the preparation of this manuscript is available upon written request from the corresponding author.

## Supplementary Materials

The following supporting information can be downloaded at: https://www.mdpi.com/article/10.3390/cells12162055/s1, Table S1: ROC analysis for CVID and CLL patients in relation to healthy volunteers.
