# Supplementary material for: Unraveling the Role of Toll-like Receptors in the Immunopathogenesis of Selected Primary and Secondary Immunodeficiencies
Source: Cells. 2023 Aug 12;12(16):2055. doi: 10.3390/cells12162055 (PMC10453926; doi:10.3390/cells12162055)
Supplement: Supplementary file 1 [file cells-12-02055-s001.zip › cells-2519560-supplementary.pdf]

Supplementary Table S1. ROC analysis for CVID nad CLL patients in relations to healthy volunteers

Parametrs		CVID vs. Healthy volunteers	CLL vs. Healthy volunteers
CD4+/TLR2+ T lymphocytes [%]	AUC	1,00	0,964
	SE	0,00	0,021
	+95%	1,00	0,924
	-95%	1,00	1,00
	Z statistic	-	22,527
	p-value	0,000*	0,000*
CD8+TLR2+ T lymphocytes [%]	AUC	1,00	1,00
	SE	0,00	0,00
	+95%	1,00	1,00
	-95%	1,00	1,00
	Z statistic	-	-
	p-value	0,000*	0,000*
CD19+TLR2+ B lymphocytes [%]	AUC	0,950	1,00
	SE	0,022	0,00
	+95%	0,906	1,00
	-95%	0,993	1,00
	Z statistic	20,201	-
	p-value	0,000*	0,000*
CD4+/TLR3+ T lymphocytes [%]	AUC	0,874	0,909
	SE	0,037	0,033
	+95%	0,802	0,845
	-95%	0,947	0,973
	Z statistic	10,119	12,526
	p-value	0,000*	0,000*
CD8+TLR3+ T lymphocytes [%]	AUC	0,842	0,821
	SE	0,043	0,048
	+95%	0,757	0,726
	-95%	0,927	0,916
	Z statistic	7,901	6,631
	p-value	0,000*	0,000*
CD19+TLR3+ B lymphocytes [%]	AUC	0,617	0,999
	SE	0,065	0,001
	+95%	0,491	0,997
	-95%	0,744	1,00
	Z statistic	1,815	469,138
	p-value	0,069	0,000*
CD4+/TLR4+ T lymphocytes [%]	AUC	0,972	1,00
	SE	0,014	0,00
	+95%	0,944	1,00
	-95%	0,999	1,00
	Z statistic	33,533	-
	p-value	0,000*	0,000*
CD8+TLR4+ T lymphocytes [%]	AUC	1,00	1,00
	SE	0,00	0,00
	+95%	1,00	1,00
	-95%	1,00	1,00

	Z statistic	-	-
	p-value	0,000*	0,000*
**CD19+TLR4+ B lymphocytes [%]**	AUC	0,338	1,00
	SE	0,061	0,00
	+95%	0,219	1,00
	-95%	0,457	1,00
	Z statistic	-2,667	-
	p-value	0,007*	0,000*
**CD4+/TLR7+ T lymphocytes [%]**	AUC	0,003	0,998
	SE	0,003	0,002
	+95%	-0,003	0,993
	-95%	0,008	1,00
	Z statistic	-167,034	211,089
	p-value	0,000*	0,000*
**CD8+TLR7+ T lymphocytes [%]**	AUC	0,011	0,880
	SE	0,007	0,037
	+95%	-0,004	0,807
	-95%	0,025	0,953
	Z statistic	-67,308	10,201
	p-value	0,000*	0,000*
**CD19+TLR7+ B lymphocytes [%]**	AUC	0,003	1,00
	SE	0,003	0,00
	+95%	-0,003	1,00
	-95%	0,008	1,00
	Z statistic	-167,034	-
	p-value	0,000*	0,000*
**CD4+/TLR8+ T lymphocytes [%]**	AUC	0,047	0,933
	SE	0,002	0,026
	+95%	-0,004	0,882
	-95%	0,086	0,984
	Z statistic	-22,580	16,71
	p-value	0,000*	0,000*
**CD8+TLR8+ T lymphocytes [%]**	AUC	1,00	0,926
	SE	0,00	0,033
	+95%	1,00	0,861
	-95%	1,00	0,991
	Z statistic	-	12,821
	p-value	0,000*	0,000*
**CD19+TLR8+ B lymphocytes [%]**	AUC	0,004	0,0993
	SE	0,0004	0,007
	+95%	-0,004	0,980
	-95%	0,012	1,00
	Z statistic	-122,904	73,201
	p-value	0,000*	0,000*
**CD4+/TLR9+ T lymphocytes [%]**	AUC	1,00	1,00
	SE	0,00	0,00
	+95%	1,00	1,00
	-95%	1,00	1,00
	Z statistic	-	-
	p-value	0,000*	0,000*

**CD8+TLR9+ T lymphocytes [%]**	AUC	1,00	1,00
	SE	0,00	0,00
	+95%	1,00	1,00
	-95%	1,00	1,00
	Z statistic	-	-
	p-value	0,000*	0,000*
**CD19+TLR9+ B lymphocytes [%]**	AUC	1,00	1,00
	SE	0,00	0,00
	+95%	1,00	1,00
	-95%	1,00	1,00
	Z statistic	-	-
	p-value	0,000*	0,000*
**Serum concentration sTLR2 [ng/ml]**	AUC	1,00	1,00
	SE	0,00	0,00
	+95%	1,00	1,00
	-95%	1,00	1,00
	Z statistic	-	-
	p-value	0,000*	0,000*
**Serum concentration sTLR3 [ng/ml]**	AUC	1,00	1,00
	SE	0,00	0,00
	+95%	1,00	1,00
	-95%	1,00	1,00
	Z statistic	-	-
	p-value	0,000*	0,000*
**Serum concentration sTLR4 [ng/ml]**	AUC	1,00	1,00
	SE	0,00	0,00
	+95%	1,00	1,00
	-95%	1,00	1,00
	Z statistic	-	-
	p-value	0,000*	0,000*
**Serum concentration sTLR7 [ng/ml]**	AUC	1,00	1,00
	SE	0,00	0,00
	+95%	1,00	1,00
	-95%	1,00	1,00
	Z statistic	-	-
	p-value	0,000*	0,000*
**Serum concentration sTLR8 [ng/ml]**	AUC	1,00	1,00
	SE	0,00	0,00
	+95%	1,00	1,00
	-95%	1,00	1,00
	Z statistic	-	-
	p-value	0,000*	0,000*
**Serum concentration sTLR9 [ng/ml]**	AUC	1,00	1,00
	SE	0,00	0,00
	+95%	1,00	1,00
	-95%	1,00	1,00
	Z statistic	-	-
	p-value	0,000*	0,000*

* Statistically significant results.
